# Serum metabolites in non-alcoholic fatty-liver disease development or reversion; a targeted metabolomic approach within the PREDIMED trial

**DOI:** 10.1186/s12986-017-0213-3

**Published:** 2017-09-02

**Authors:** Christopher Papandreou, Mònica Bullò, Francisco José Tinahones, Miguel Ángel Martínez-González, Dolores Corella, Georgios A. Fragkiadakis, José López-Miranda, Ramon Estruch, Montserrat Fitó, Jordi Salas-Salvadó

**Affiliations:** 1 Human Nutrition Department, Hospital Universitari Sant Joan, Institut d’Investigació Sanitaria Pere Virgili, Universitat Rovira i Virgili, Reus, Spain; 2 Ciber Fisiopatología de la Obesidad y Nutrición (CIBEROBN), Instituto de Salud Carlos III (ISCIII), Madrid, Spain; 3 Unidad de Gestión Clínica de Endocrinología y Nutrición, Instituto de Investigación Biomédica de Málaga (IBIMA), Hospital Clínico Virgen de la Victoria/Universidad de Málaga, Malaga, Spain; 4 Department of Preventive Medicine and Public Health, School of Medicine, University of Navarra, Pamplona, Spain; 5 Department of Preventive Medicine, University of Valencia, Valencia, Spain; 6 Department of Nutrition & Dietetics, Technological Education Institute of Crete, Crete, Greece; 7 Lipid and Atherosclerosis Unit, Department of Internal Medicine, Reina Sofia University Hospital, IMIBIC, University of Cordoba, Cordoba, Spain; 8 Department of Internal Medicine, Hospital Clínic, IDIBAPS, Barcelona, Spain; 9 Cardiovascular and Nutrition Research Group, Institut de Recerca Hospital del Mar, Barcelona, Spain; 10 Human Nutrition Unit, Faculty of Medicine and Health Sciences, Universitat Rovira i Virgili, St/Sant Llorenç 21, 43201 Reus, Spain

**Keywords:** Non-alcoholic fatty liver disease, Metabolomics, Fatty acid metabolism, Hepatic lipotoxicity

## Abstract

**Background:**

Limited prospective studies have examined changes in non-alcoholic fatty-liver disease (NAFLD) related serum-metabolites and none the effects of NAFLD-reversion. We aimed to evaluate whether perturbations in metabolites indicate predisposition to NAFLD development and to assess the effects of NAFLD reversion on metabolite profiles.

**Methods:**

A targeted liquid-chromatography tandem mass-spectrometry metabolic profiling (*n* = 453 metabolites) approach was applied, using serum from 45 subjects of the PREDIMED study, at baseline and after a median 3.8-year follow-up. NAFLD was determined using the hepatic steatosis index; with three groups classified and studied: Group 1, not characterized as NAFLD cases during the follow-up (*n* = 15); Group 2, characterized as NAFLD during the follow-up (*n* = 15); Group 3, characterized as NAFLD-reversion during the follow-up (*n* = 15).

**Results:**

At baseline, significantly lower storage and transport lipids (triacylglycerols and cholesteryl esters), several monoetherglycerophosphocholines, acylglycerophosphocholines, ceramides and ceramide to sphingomyelin ratio (*P* < 0.05), were found; whereas a higher L-cystine to L-glutamate ratio (*P* < 0.05) was observed, in group 2 as compared to group 1.P-ether acylglycerophosphocholines, ceramides and sphingolipids were significantly different betweengroup 3 and group 1 (*P* < 0.05). Higher 16:1n-7 to 16:0, and 18:0 to16:0 ratio (*P* < 0.05), while lower 18:1n-9 to 18:0, 16:0 to 18:2n-6, and 18:3n-6 to 18:2n-6 ratio (*P* < 0.05) were observed in the final, compared to baseline values, in groups 2 and 3.

**Conclusion:**

The rearrangement of lipid biosynthesis and serum transport may indicate predisposition to NAFLD development. Despite an expected reduction of hepatic lipotoxicity and improved hepatic function in the participants of the study characterized as NAFLD-reversing, the side effects of NAFLD in serum metabolic profiles remained present.

**Trial registration:**

The trial is registered at ISRCTN35739639. Registration date: 5th October 2005.

**Electronic supplementary material:**

The online version of this article (doi:10.1186/s12986-017-0213-3) contains supplementary material, which is available to authorized users.

## Background

Non-alcoholic fatty liver disease (NAFLD) is common worldwide, with a prevalence of 6–35% [1–3] and includes a spectrum of pathophysiology stages, developing from simple steatosis to non-alcoholic steatohepatitis (NASH) and cirrhosis. NAFLD, characterized by lipid accumulation within hepatocytes, is a major cause of liver-related morbidity and mortality [4]. Although NAFLD pathogenesis is not yet fully understood, progress has been made in elucidating the mechanisms involved on its development [5], in which obesity and insulin resistance may play an important role [6]. Consequently, the development of NAFLD is accompanied by metabolic changes and alterations in body lipids metabolism [7].

Recently, several studies focused on NAFLD association with metabolism of complex lipid classes. Following this approach, Puri et al. [8] reported perturbations in hepatic lipid classes, during progression from normal liver status to steatosis and NASH, as accumulation of triacyl- and diacyl-glycerols, free cholesterol, cholesterol esters and saturated fatty acids [8]. Concerning phospholipids especially, their composition appears to differentiate in the liver of patients with steatosis and NASH, with decreased concentrations of phosphatidylcholine and phosphatidylethanolamine in steatosis [8]. Concerning plasma, increased concentrations of glycerolphosphocholines, glycerolphosphoethanolamines, glycerolphosphoinositols, glycerolphosphoglycerols, lysoglycerophosphocholines, and ceramides were reported in NASH patients [9]. In addition, decreased plasma polyunsaturated fatty acids (PUFAs), as well as increased monounsaturated fatty acids (MUFAs) and eicosanoid metabolites of the lipoxygenase pathway have been reported in plasma of NAFLD patients [10].

Regarding enzymes regulating fatty acid metabolism, a higher activity of delta-6 desaturase and stearoyl-CoA desaturase-1 and lower activity of delta-5 desaturase is reported in steatosis compared to non-steatosis [11], and in NASH compared to non-NASH, independently of obesity indexes and diet [12]. Besides lipids, several essential amino acids, branched chain amino acids (BCAAs) and phenylalanine, as well as the non-essential amino acids aspartate and glutamate, have been found elevated in plasma samples of NASH patients but not in steatosis, possibly indicating a higher rate of whole body protein turnoverin NASH possibly due to inflammation combined with insulin resistance [13, 14].

The metabolic complexity of NAFLD presents challenges for a deeper understanding of the metabolic pathways that contribute to the development of this pathology. Progress in this area can be aided by comprehensive metabolic analysis of the condition before or after the disease is established [15, 16]. To the best of our knowledge, there are limited prospective studies examining changes in NAFLD-related metabolites in human plasma or serum and no previous studies have examined metabolic profiles of NAFLD-reversion. This analysis may facilitate the development of early or/and continuous monitoring for potential interventions aimed at preventing/reversing the disease.

Taking the above into account, the present study aimed to evaluate whether perturbations in serum-metabolites can indicate overall predisposition to NAFLD development in participants of the PREDIMED (Prevention of Disease with Mediterranean Diet) study. Furthermore, the differences in baseline metabolites between the groups of participants characterized as NAFLD or not, as well as the changes in the metabolite profiles in the group of participants characterized as NAFLD-reversion, were analysed.

## Methods

### Study design and participants

This study is a prospective design sub-study within the PREDIMED trial (ISRCTN35739639). The design of the PREDIMED trial has been described in detail elsewhere [17, 18]. In brief, 7447 participants at high cardiovascular risk and free of severe chronic illness including virus hepatitis, drug or alcohol addiction, were randomly assigned to a Mediterranean-diet regime, supplemented with extra-virgin olive oil; a Mediterranean diet supplemented with mixed nuts, or a control diet consisting of advice to reduce the fat intake. The Institutional Review Boards of the recruitment centers approved the study protocol, and participants gave their informed consent. For the present study, 2543 participants assigned to the Mediterranean diet supplemented with extra-virgin olive oil were assessed for the presence of NAFLD at baseline and during the follow-up. For the needs of this study, the presence of NAFLD was determined using two criteria: I). A hepatic steatosis index (HSI) ≥ 36 for both genders. The HSI is a simple index used to predict the presence of NAFLD with reasonable accuracy [19]. II). Alcohol intake <210 g/week for men and <140 g/week for women. Three Groups were formed each consisting of 15 individuals randomly selected from 2543 participants and not using any medication affecting the transaminase levels (except for statins) Group 1: Fifteen participants did not meet the NAFLD criteria we used, at baseline and during the follow-up (cases not characterized as NAFLD); Group 2: Fifteen participants did not meet NAFLD criteria at baseline, but met NAFLD criteria during the follow-up (cases characterized as NAFLD); Group 3: Fifteen participants met NAFLD criteria at baseline, but not during the follow-up (cases characterized as NAFLD-reversion). Person time of follow-up was calculated as the interval between the randomization date and the earliest date of the follow-up contact at which a new characterization of NAFLD or NAFLD reversion was ascertained, death from any cause, or date of the last contact visit, whichever came first. The median participant’s follow-up was 3.8 years (minimum, 11 months; maximum, 6.8 years). The three groups were matched for time of follow-up (<3 years, between 3 and 6 years and between 6 and 9 years), age (≤65 vs. > 65 y), gender, and body-mass-index (≤26 vs. > 26 kg/m^2^).

### Targeted metabolomics approach to quantify metabolites

Fasting serum samples were collected from subjects and stored at −80 °C. Metabolomic analyses were run by paired samples for each participant (baseline and at the end of the follow-up). Because in metabolic profiling there is no single platform or method to analyse the entire metabolome of a biological sample mainly due to the wide concentration range of the metabolites, combined to their extensive chemical diversity [20, 21], the current study used multiple liquid chromatography tandem mass spectrometry (LC-MS/MS) platforms) based on a Waters QTOF Premier instrument (Waters Corp., Milford, MA), optimized for extensive coverage of the serum metabolome [22]. Three separate LC-MS/MS based platforms were used to perform optimal profiling of: (1) Fatty acyls, bile acids, steroids and lysoglycerophospholipids; (2) glycerolipids, glycerophospholipids, sterol lipids and sphingolipids; (3) amino acids. As described by Barr et al. [23], the following methods were used according to the target analytes’ chemical class. This section and the pre-processing of metabolomics data are detailed in the Additional file 1. Two different types of quality control (QC) samples were used to assess the data quality for all the aforementioned platforms [24].

### Estimation of index of enzyme activities

Index of enzyme activities were estimated by calculating the product-to-precursor ratios of individual fatty acids (FAs) according to previous studies [12, 25]. More details can be found in the Additional file 2.

### Statistical analysis

Data on general characteristics of the study population are presented as means (± standard deviations) for continuous variables and percentages for categorical variables. Differences in these characteristics among the three groups were tested by using one-way analysis of variance (ANOVA) with Bonferroni correction for multiple comparisons or the Kruskal–Wallis as appropriate, in the case of continuous variables and chi-squared test in the case of categorical variables. ANOVA with Turkey’s HSD (Honestly Significance Difference) post hoc test was performedfor the comparison in the levels of metabolites between the three baseline states of the three groups, while a paired Student’s t-test was carried out for the comparisons between baseline and final states in each group. The level of statistical significance was set at *p* < 0.05.

The principal component analysis (PCA) [26] was applied to identify outliers and detect data grouping or separation trends. The quality of the PCA models was described by R^2^ and Q^2^ values. R^2^ is defined as the proportion of variance in the data explained by the models and indicates goodness of fit, and Q^2^ is defined as the proportion of variance in the data predicted by the model and indicates predictability. Orthogonal partial least-squares to latent structures (OPLS) approach [27, 28] was used to separate the systematic variation among the metabolites that is related to group separation. The goodness of fit parameters for the OPLS model, R^2^X, R^2^Y and goodness of prediction Q^2^Y were calculated, varying from 0 to 1. R^2^X and R^2^Y represent the fraction of the variance of the X and Y variable explained by the model, while Q^2^Y suggests the predictive performance of the model. Multivariate statistical analyses were performed using SIMCA 14.1.0 software (Umetrics, Umeå, Sweden) while univariate analyses were performed using the R statistical package (version 3.1.1; R Development Core Team, 2011; http://cran.r-project.org).

## Results

The general characteristics of study subjects (*n* = 45) are shown in Table 1. No significant differences between the three groups were observed in relation to the statins use. At baseline, the three groups were similar with respect to most characteristics with the exception (Table 1) of aspartate transaminase (AST) and aspartate transaminase to alanine transaminase ratio (AST/ALT), which decrease in the group of those not characterized as NAFLD and increase in the NAFLD-characterized group (*p* < 0.05).

**Table 1:** Characteristics of studied groups

Characteristics	Group 1 (*n* = 15)			Group 2 (*n* = 15)			Group 3 (*n* = 15)		
	Basal	Final	Change	Basal	Final	Change	Basal	Final	Change
Age (years)	63.47 ± 6.59	NA	NA	65.13 ± 5.74	NA	NA	64.07 ± 6.94	NA	NA
MedScore	9.73 ± 1.83	10.31 ± 1.88	0.31 ± 2.81	9.43 ± 2.10	11.43 ± 1.22	1.85 ± 1.72	9.53 ± 2.16	10.47 ± 1.59	0.93 ± 2.49
Alcohol (g)	16.30 ± 24.10	10.77 ± 11.63	−5.70 ± 15.71	29.93 ± 28.58	12.75 ± 10.71	−12.89 ± 24.40^b,c^	7.48 ± 10.11	11.76 ± 13.07	4.28 ± 6.82
BMI (kg/m^2^)	27.36 ± 2.10	27.21 ± 3.38	−0.08 ± 2.04	28.65 ± 3.39	29.68 ± 3.72	0.91 ± 1.94	29.08 ± 2.84	28.18 ± 3.12	−0.90 ± 1.98
WC (cm)	97.29 ± 9.41	99.09 ± 12.11	1.30 ± 6.11	98.43 ± 10.28	102.07 ± 11.82	2.23 ± 7.86	100.80 ± 11.69	99.20 ± 12.52	−1.60 ± 7.33
AST (U/L)	20.36 ± 5.84	17.82 ± 6.70^a,b^	−0.91 ± 6.93	21.14 ± 6.51	26.14 ± 8.20^b,c^	5.00 ± 7.68	21.60 ± 11.41	19.00 ± 6.03	−2.60 ± 14.12
AST/ALT	0.61 ± 0.28^a,b/a,c^	0.49 ± 0.19^a,b/a,c^	−0.08 ± 0.20	0.80 ± 0.23	0.88 ± 0.16	0.08 ± 0.18^b,c^	0.97 ± 0.25	0.84 ± 0.37	−0.13 ± 0.26
Glu (mg/dL)	131.33 ± 49.75	108.16 ± 19.73	−18.50 ± 38.10	124.97 ± 42.31	122.07 ± 59.13	−2.90 ± 61.46	138.38 ± 46.72	156.53 ± 62.44	18.14 ± 54.08
TG (mg/dL)	158.33 ± 81.12	158.00 ± 51.73	4.83 ± 53.32	117.98 ± 53.23	160.78 ± 98.12	42.80 ± 78.71	157.54 ± 106.38	150.13 ± 110.68	−7.41 ± 137.09
TC (mg/dL)	229.43 ± 49.23	201.85 ± 41.16	−30.68 ± 55.85	207.64 ± 46.53	195.21 ± 25.70	−12.43 ± 45.39	208.07 ± 45.39	190.15 ± 36.21	−17.92 ± 36.83
HDL-C (mg/dL)	49.26 ± 11.86	45.90 ± 12.29	−3.32 ± 10.33	56.38 ± 12.54	53.71 ± 11.21	−2.67 ± 10.04	48.41 ± 8.82	55.74 ± 16.84	6.52 ± 14.54
LDL-C (mg/dL)	132.97 ± 28.29	114.67 ± 32.60	−20.05 ± 26.78	127.79 ± 39.65	110.03 ± 21.69	−17.77 ± 48.77	121.14 ± 33.85	105.45 ± 29.71	−15.22 ± 23.65
Diabetes	40.0%	NA	NA	60.0%	NA	NA	60%	NA	NA
Hypercholest	66.7%	NA	NA	66.7%	NA	NA	46.7%	NA	NA
Hypertension	93.3%	NA	NA	80.0%	NA	NA	80%	NA	NA

Data are expressed as means ± SD or percentage

One-way analysis of variance (ANOVA) or Kruskal-Wallis test for continuous variables and Pearson Chi-Square test for categorical variables

Microalbuminuria changes in characteristics of studied groups at the end of the follow-up were excluded from analysis, because there were only few cases, namely 18 out of 45

Abbreviations: *NA* Not Available, *MedScore* Mediterranean Diet Score, *BMI* Body Mass Index, *WC* Waist Circumference, *AST* aspartate aminotransferase, *Glu* Glucose, *TC* Total cholesterol, *HDL-C* High-density lipoprotein cholesterol, *LDL-C* Low-density lipoprotein cholesterol, *Hypercholest* Hypercholesterolemia

^a,b,c^: Identical superscript letters denote significant differences between groups (*p* < 0.05 after *post-hoc* multiple comparisons using the Bonferroni rule in the case of normally distributed continuous variables, Mann-Whitney test in the case of non- normally distributed continuous variables)

Group 1 Participants did not meet NAFLD criteria at the baseline and during the follow-up

Group 2 Participantsdid not meet NAFLD criteria at baseline, but met NAFLD criteria during the follow-up

Group 3 Participants met NAFLD criteria at baseline, but not during the follow-up

^a,b^p Value <0.05 “Group 1” vs. “Group 2”

^a,c^ *p* Value <0.05 “Group 1” vs. “Group 3”

^b,c^ *p* Value <0.05 “Group 2” vs. “Group 3”

A total of 453 distinct metabolites were detected in the analysed serum samples and included in the subsequent multivariate and univariate data analyses. An initial unsupervised PCA did not result in any clear discrimination between the basal states per se between the three groups (Additional file 3: Fig. S1) and their baseline and the final states (Additional files 4, 5 and 6: Figs. S2-S4). The subsequent supervised OPLS discriminator analysis showed a uniform overlap between the aforementioned groups and baseline and final states, presenting non-significant prediction values (data not shown).

### Univariate analysis of the three baseline states

Differences in the content of metabolites between the three baseline states were visualized through a heat map (Additional file 7: Fig. S5). On the left-hand side of this Figure the ANOVA is displayed, and then the log_2_ (fold change) of the metabolites and Tukey’s HSD post hoc test for each mentioned comparisons are indicated. The Tukey’s HSD post-hoc test analyses performed per each metabolic class and ratios were performed and the significant results are shown in Table 2.

**Table 2:** Comparison of metabolites concentrations and enzyme activities based on fatty acid ratios between the three baseline states in groups 1, 2 and 3

		Baseline 3 vs. Baseline 2		Baseline 3 vs. Baseline 1		Baseline 2 vs. Baseline 1	
	ANOVA (p)	Tukey’s HSD (*p*-value)	log_2_ (fold change)	Tukey’s HSD (*p*-value)	log_2_ (fold change)	Tukey’s HSD (*p*-value)	log_2_ (fold change)
Triacylglycerols	**4.28E-02**	9.78E-01	0.06	9.18E-02	−0.49	5.95E-02	−0.55
MonoetherglycerophosphocholineO_plasmenyles	**4.28E-03**	**4.70E-03**	0.39	7.45E-03	0.08	**3.18E-02**	−0.32
MonoetherglycerophosphocholineP_plasmenyles	**5.30E-03**	**1.79E-02**	0.44	9.66E-01	−0.03	**9.35E-03**	−0.47
1-Monoetherglycerophosphocholine	**1.70E-03**	**3.70E-03**	0.41	9.77E-01	0.02	**6.55E-03**	−0.39
1-ether, 2-acylglycerophosphocholine O_plasmenyles	**1.50E-02**	5.56E-01	0.14	1.34E-01	−0.23	**1.22E-02**	−0.37
1-ether, 2-acylglycerophosphocholine P_plasmenyles	**6.77E-03**	9.07E-01	0.09	**2.80E-02**	−0.46	**9.56E-03**	−0.55
1-ether, 2-acylglycerophosphocholines	**7.19E-03**	6.95E-01	0.12	5.43E-02	−0.31	**7.06E-03**	−0.43
MonoetherglycerophosphoethanolaminesP_plasmenyles	**4.18E-03**	9.61E-02	0.41	9.65E-01	−0.04	5.54E-02	−0.46
Ceramides	**1.05E-03**	9.81E-01	−0.03	**2.51E-03**	−0.45	**4.29E-03**	−0.42
N-acyl ceramides	**3.99E-03**	9.94E-01	0.01	**1.15E-02**	−0.37	**8.77E-03**	−0.39
N-acyl sphingosines	**3.99E-03**	9.94E-01	0.01	**1.15E-02**	−0.37	**8.77E-03**	−0.39
Total sphingolipids	**3.03E-02**	7.81E-01	−0.10	**2.95E-02**	−0.36	1.29E-01	−0.26
Sum of Triacylglycerols& Cholesteryl Esters	**2.34E-02**	9.94E-01	0.03	**5.00E-02**	−0.49	**3.92E-02**	−0.51
L-Cystine to L-Glutamate	**3.41E-02**	9.61E-01	−0.06	8.30E-02	0.60	**4.58E-02**	0.66
Sphingomyelinase (Cer/SM)	**2.16E-02**	5.69E-01	0.09	1.68E-01	−0.15	**1.76E-02**	−0.23

log_2_ (fold-change) and *p*-values follows the criterion of heatmap figure

One-way analysis of variance (ANOVA) and Tukey’s Honestly Significance Difference (HSD) post hoc test

Bold indicates statistically significant *p*-values

Group 1 Participants did not meet NAFLD criteria at the baseline and during the follow-up

Group 2 Participants did not meet NAFLD criteria at baseline, but met NAFLD criteria during the follow-up

Group 3 Participants met NAFLD criteria at baseline, but not during the follow-up

Abbreviations: *Cer* ceramides, *SM* sphingomyelin, *ChoE* cholesteryl esters, *Chol* cholesterol

At baseline, there was not found a significant difference in serum TAGs (*p* = 0.059) or the sum of TAGs and cholesteryl esters (*p* = 0.039) between the cases characterized as NAFLD, compared to non-characterized NAFLD cases. In addition, the levels of several monoether-glycero-phosphocholines and acyl-glycero-phosphocholines (*p* < 0.05) were lower in the group of the characterized as NAFLD cases. Lower levels of ceramides (*p* = 4.2 × 10–3) were also found at baseline in these participants compared with those not-characterized as NAFLD. The activity index of sphingomyelinase (Cer/SM) (*p* = 0.018) was lower, while the L-cystine to L-glutamate ratio (*p* = 0.046) was higher at baseline in the characterized as NAFLD cases.

At baseline, those participants characterized as NAFLD-reversing, compared to non-suspected cases, revealed lower serum levels in P-ether acyl-glycero-phosphocholines (*P* = 0.028), ceramides (*p* = 0.002), total sphingolipids (*p* = 0.029) but not significantly lower serum levels of the sum of TAGs and cholesteryl esters (*p* = 0.05). Higher baseline values of several mono-ether-glycero-phosphocholines (*p* < 0.05) were found in those participants reversing NAFLD than those who met NAFLD criteria during the follow-up. No significant baseline differences in other lipid classes or index of enzyme activities were observed.

### **Univariate analysis comparing final** vs.**baseline states**

A heatmap visualising the differences in metabolite concentrations between the final state and the baseline state of each group is shown in Additional file 8: Fig. S6. It displays the log_2_ (fold-change) of the metabolites included in the analysis together with the Student’s t-test for all the comparisons performed. Additionally, volcano plots (*p*-value versus fold-change) were generated for the aforementioned comparisons (Additional files 9, 10 and 11: Figs. S7-S9), complementing the results presented in the heatmap.

In group 1 (cases not-characterized as NAFLD), lower levels of monoacylglycerophosphocholines and monoetherglycerophosphocholine (*p* < 0.05), monoetherglycerophosphoethanolamines (*p* = 0.002) were found at the end of the follow-up compared with baseline values (Table 3). Lower levels of lysophosphatidylcholines (*p* = 0.015), TAGs (*p* = 0.036) and sum of TAGs and cholesteryl esters (*p* = 0.025) and a higher index of SCD1 activity (16:1n-7/16:0) (*p* = 0.045) were observed in final samples.

**Table 3:** Comparison of metabolites concentrations and enzyme activities based on fatty acid ratios between the final and the baseline status (Final vs. Baseline) in group 1 (not meeting the NAFLD criteria at baseline and during the follow-up)

Metabolites and enzyme activities	Paired Student’s t-test (*p*-value)	log_2_ (fold change)
1-Monoacylglycerophosphocholine	3.26E-02	−0.19
2-Monoacylglycerophosphocholine	3.59E-02	−0.20
Total Monoacylglycerophosphocholine	3.35E-02	−0.19
MonoetherglycerophosphocholineP_plasmenyles	3.60E-02	−0.20
MonoetherglycerophosphoethanolaminesO_plasmenyles	2.05E-03	−0.64
Total 1- Monoetherglycerophosphocholine	2.92E-02	−0.18
Total Lysophosphatidylcholines	1.57E-02	−0.21
Triacylglycerols	3.68E-02	−0.23
Sum of Triacylglycerols& Cholesteryl Esters	2.50E-02	−0.27
Stearoyl-CoA desaturase 1 (16:1n-7/16:0)	4.53E-02	0.09

log_2_ (fold-change) and *p*-values follows the criterion of heatmap figure

Significantly lower levels of SFA (*p* = 0.016), monoacylglycerophosphocholines (*p* < 0.050) and N-Acyl ethanolamines (*p* = 0.024) were found in group 2 (cases characterized as NAFLD) at the end of the follow-up compared with baseline (Table 4). Higher index of elongase 6 (18:0/16:0) (*p* = 0.031), D6D (18:4n-3/18:3n-3) (*p* = 0.012), SCD1 (16:1n-7/16:0) (*P* = 0.011) activities, a higher PUFA to MUFA ratio (*p* = 0.015), and MUFA + PUFA to saturated FA (SFA) ratio (*p* = 7.5 × 10^−5^), as well as a lower non esterified fatty acids (NEFA) n-6 to n-3 ratio (*p* = 0.037) were found in final samples compared to baseline values. On the other hand, lower index of SCD1 (18:1n-9/18:0) (*p* = 0.017), D6D [18:3n-6/18:2n-6 (*p* = 0.002) and 18:3/18:2) (*p* = 0.007)] activities were observed. The ratios of MAPC to MAPE and LPC to LPE were also lower in final samples (*p* = 8.2 × 10^−4^ and *p* = 5.0 × 10^−4^ respectively). Finally, compared to baseline values, an index of de novo lipogenesis (16:0/18:2n-6) (*p* = 0.002) was significantly lower at the end.

**Table 4:** Comparison of metabolites concentrations and enzyme activities based on fatty acid ratios between the final and the baseline status (Final vs. Baseline) in group 2 (not meeting the NAFLD criteria at baseline but meeting them during the follow-up)

Metabolites and enzyme activities	Paired Student’s t-test (*p*-value)	log_2_ (fold change)
Steroids	9.05E-03	−0.38
Saturated fatty acids	1.64E-02	−0.42
Primary fatty amides	4.26E-02	−0.34
N-Acyl ethanolamines	2.39E-02	−0.23
1-Monoacylglycerophosphocholine	2.78E-02	−0.16
Monoacylglycerophosphocholine	4.00E-02	−0.14
Desaturase (MUFA + PUFA/SFA)	7.55E-04	0.39
MAPC/MAPE	8.21E-04	−0.34
LPC/LPE	5.71E-04	−0.31
(NEFA) n-6 /n-3	3.69E-02	−0.27
De novo lipogenesis (16:0/18:2n-6)	1.72E-03	−0.48
Stearoyl-CoA desaturase 1 (18:1n-9/18:0)	1.79E-02	−0.33
Elongase-6 (18:0/16:0)	3.13E-02	0.70
Delta-6- desaturase (18:3n-6/18:2n-6)	2.39E-03	−0.48
Delta-6- desaturase (18:3/18:2)	7.64E-03	−0.39
Delta-6- desaturase(18:4n-3/18:3n-3)	1.21E-02	2.72
Stearoyl-CoA desaturase 1 (16:1n-7/16:0)	1.11E-02	0.11
18:1/18:0	2.69E-02	−0.12
PUFA/MUFA	1.50E-02	0.25

log_2_ (fold-change) and *p*-values follows the criterion of heatmap figure

Abbreviations: *SFA* saturated fatty acids, *PUFA* polyunsaturated fatty acids, *MUFA* monounsaturated fatty acids, *MAPC* Monoacylglycerophosphocholine, *MAPE* Monoacylglycerophosphoethanolamine, *LPC* lysophosphatidylcholine, *LPE* lysophosphatidylethanolamine, *NEFA* non esterified fatty acids

In group 3 (NAFLD-reversion cases), compared to baseline values, SFA (*p* = 0.013), monoacylglycerophosphocholines (*p* < 0.05), monoacylglycerophosphoethanolamine (*p* = 0.012), monoetherglycerophosphoethanolamines (*p* = 0.002) and lysophosphatidylcholine (*p* = 0.015) were lower at the end of follow-up (Table 5). Similarly to the characterized cases of NAFLD, higher 16:1n-7 to 16:0 (*p* = 0.003) and lower 18:1n-9 to 18:0 (*p* = 3.6 × 10^−4^) ratios were observed at the end, while the index of elongase 6 activity (18:0/16:0) was higher (*p* = 6.7 × 10^−5^). Compared to baseline values, at the end of follow-up a higher ratio of NEFA n-6 to n-3 (*p* = 0.011) and the index of lecithin: cholesterol acyltransferase activity (ChoE/Chol) (*p* = 0.026) was found, while the index of lower de novo lipogenesis (16:0/18:2n-6) (*p* = 8.4 × 10^−4^) and D6D activity (18:3n-6/18:2n-6) (*p* = 4.6 × 10^−4^) has been demonstrated. The ratios of LPC to PC (*p* = 0.008) and LPE to PE (*p* = 0.019) were also found lower at the end of follow-up.

**Table 5:** Comparison of metabolites concentrations and enzyme activities based on fatty acid ratios between the final and the baseline status (Final vs. Baseline) in group 3 (meeting the NAFLD criteria at baseline but not meeting them during the follow-up)

Metabolites and enzyme activities	Paired Student’s t-test (*p*-value)	log_2_ (fold change)
Steroids	1.99E-02	−0.46
Saturated fatty acids	1.36E-02	−0.30
1-Monoacylglycerophosphocholine	2.15E-03	−0.27
2-Monoacylglycerophosphocholine	2.85E-02	−0.16
Monoacylglycerophosphocholine	1.16E-02	−0.20
Lysophosphatidylcholines	1.49E-02	−0.17
1-Monoacylglycerophosphoethanolamine	1.23E-02	−0.23
MonoetherglycerophosphoethanolaminesO_plasmanyles	2.12E-02	−0.32
Phospholipase-A2 (LPC/PC)	8.38E-03	−0.28
Phospholipase-A2 (LPE/PE)	1.91E-02	−0.21
(NEFA) n-6/n-3	1.15E-02	0.57
De novo lipogenesis (16:0/18:2n-6)	8.49E-04	−0.43
Stearoyl-CoA desaturase 1 (18:1n-9/18:0)	2.35E-04	−0.58
Elongase-6 (18:0/16:0)	6.78E-05	0.71
Delta-6- desaturase (18:3n-6/18:2n-6)	4.65E-04	−0.63
Delta-6- desaturase (18:3/18:2)	2.43E-04	−0.55
Stearoyl-CoA desaturase 1 (16:1n-7/16:0)	3.22E-03	0.11
18:1/18:0	3.65E-04	−0.21
Lecithin:cholesterolacyltransferase (ChoE/Chol)	2.64E-02	0.47

log_2_ (fold-change) and *p*-values follows the criterion of heatmap figure

Abbreviations: *LPC* lysophosphatidylcholine, *LPE* lysophosphatidylethanolamine, *NEFA* non esterified fatty acids, *ChoE* cholesteryl esters, *Chol* cholesterol

No significant differences in the concentration of amino acids between the three baseline statesand between the final and the baseline state of each group were found.

## Discussion

In this study we detected differences in serum lipid classes between participants who were characterized as NAFLD and those not characterized as NAFLD after a median 3.8 years of follow-up. These differences may be an early indicator of a rearrangement of lipid biosynthesis in the liver in those participants who met NAFLD criteria during the follow-up.

The lower levels of glycerophosphocholines in addition to ceramides and the possible lower activity of sphingomyelinase could indicate a rearrangement of lipid biosynthesis in the liver in the group of cases characterized as NAFLD [29, 30]. We also observed a higher L-cystine to L-glutamate ratio in the participants meeting NAFLD criteria during the follow-up. Since higher plasma L-cystine/L-glutamate ratio was positively associated with TNF-alpha circulating levels in patients with advanced cirrhosis [31], our results may indicate an immune dysfunction state in these participants [32].

In contrast, the results of our study are not in accordance with findings from previous studies regarding differences in metabolites and enzyme activities between NAFLD and no-NAFLD [8, 10, 12, 33], possibly due to differences in methods for detecting NAFLD, in participants’ characteristics and metabolites analyses procedures. A decrease in very low-density lipoprotein particles secretion from the liver, as it has been shown to occur in NAFLD, could explain the reduction in etherglycerophosphocholines and sphingolipids in the serum of our study participants. We also did not find elevated levelsof BCAAs or other amino acids in cases characterized as NAFLD, which is in agreement with the results from previous studies examining the role of hepatic steatosis in amino acids profiles [13, 14].

Interestingly, the comparison between the group with NAFLD-reversion cases and the group with cases characterized as NAFLD did not reveal notable differences in the levels of most metabolites except for phospholipids catabolic intermediates and therefore we can imply that the hepatic lipid metabolism was similar in both groups. Besides the findings from baseline comparisons, we also found differences in the levels of metabolites and estimated enzyme activities at the end of a long-term follow-up that may indicate potential influences of NAFLD idiopathic conditions.

Research suggests that FFA-mediated cytotoxicity is indirect, via the generation of the toxic metabolite lysophosphatidylcholine (LPC) [34, 35]. A reduction in LPC content in erythrocyte membranes was observed after 1-year intervention with Mediterranean diet enriched in extra virgin olive oil in participants with similar characteristics to our study [36]. Likewise, the lower LPC levels found in our study in cases not characterized as NAFLD could be attributed to the potential effect of the diet per se, resulting in a possible reduction of hepatic lipotoxicity. Notably, in cases characterised as NAFLD, the ratio of lysophosphatidylcholine to lysophosphatidylethanolamine (LPC/LPE) was lower at the end of the follow-up. Previous research, using mouse models, suggests that the lower phosphatidylcholine to phosphatidylethanolamine (precursors of the LPC and LPE) ratio may lead to a disruption in membrane integrity, resulting in hepatocyte damage [37]. In our study, ethanolamine- and choline-glycerophospholipids, were lower at the end of the follow-up in those participants that did not meet NAFLD criteria or appeared to reverse NAFLD, while only glycerophosphocholines was lower in those who met NAFLD criteria during follow-up.

Differences in indices of enzyme activities regulating endogenous metabolism of fatty acids were observed before and at the end of the follow-up, particularly in cases characterized as NAFLD or NAFLD-reversion, indicating that NAFLD might play a prominent role in the regulation of these enzyme activities. On the other hand, in the non-NAFLD cases, the Mediterranean diet per se might have a less significant role, since only the 16:1n-7 to 16:0 ratio was found elevated. In this context, the Mediterranean diet enriched with olive oil may have up-regulated the expression of the hepatic SCD1 [38] which catalyses the biosynthesis of MUFAs from SFA.

The enzyme SCD1 catalyses the synthesis not only of 16:1n-7 but also 18:1n-9 from its precursor 18:0 [39]. The synthesis of 18:1n-9 decreased while that of 16:1n-7 increased in the cases characterized as NAFLD or NAFLD-reversion. Two previous studies also revealed an increment in the 16:1n-7 to 16:0 ratio in individuals with NAFLD, compared to those with normal liver function [10, 12]. Interestingly, a possible lower de novo lipogenesis (16:0 to 18:2n-6) may have occurred in cases characterized as NAFLD or NAFLD-reversion. A parallel activation of de novo lipogenesis and SCD1 activity has been found after 3 day of high-carbohydrate feeding in healthy subjects [40], and recently [12] was suggested as a mechanism for the elevated proportion of 16:1n-7 found in NAFLD. However, there is some evidence to suggest that de novo lipogenesis and SCD1 can be regulated independently [41]. According to Emken [42] the reduced synthesis of 18:1n-9 might be attributed to the low capacity of the liver to desaturate 18:0. On the other hand, a previous study comparing NAFLD with no-NAFLD subjects suggested a greater utilization of 18:0 along the SCD1 pathway in NAFLD rather than elongase pathway [10]. Interestingly in the groups, characterised as NAFLD or NAFLD-reversion, an increase in the ratio 18:0 to 16:0 was observed, indicating a potential increase in the FA elongase 6 (Elovl6) enzyme activity. According to a previous study the Elovl6- mediated conversion to 18:0 is a key step to hepatic lipotoxicity [43]. Additionally, the decrease in the ratio 18:1n-9 to 18:0 and increase in the 18:0 to 16:0 ratio may have led to an accumulation of 18:0 which is cytotoxic [43]. Therefore, the lower production of 16:0 due to a decrease in de novo lipogenesis combined with an increase in 16:1n-7 to 16:0 and 18:0 to 16:0 ratios may constitute an adaptive mechanism in the prevention of further liver damage by decreasing the amount of 16:0 [44]. NAFLD has also been associated with higher D6D (18:3n-6/18:2n-6) [11]. Actions of D6D, usually uses 18:2n-6 or 18:3n-3 as its natural substrate. Long-chain n-3FA levels are found decreased in the hepatic tissue of patients with NAFLD [45]. In the group, with cases characterised as NAFLD, it may be that homeostatic mechanisms were activated, to resist against the disease by “channelling” the PUFA biosynthesis routes towards long-chain n-3FA production.

Interestingly, lower LPC levels and lower LPC to PC ratio indicating a possible reduced activity of phospholipase A2 and higher lecithin: cholesterol acyltransferase activity were found during the follow-up in NAFLD-reversion, contributing to a possible reduction of hepatic lipotoxicity [34, 35], and improved hepatic function [46], respectively. However, the comparison between the final and baseline states in NAFLD-reversing and NAFLD cases, revealed similar results concerning other metabolites and particularly enzyme activities. Furthermore, the higher ratio of NEFA n-6/n-3 found in the final samples of NAFLD-reversing cases is similar to previous findings in NAFLD patients [47]; side effects of NAFLD still appear in those participants reversing it, affecting hepatic lipid metabolism.

The lack of clustering and significant prediction values after multivariate analysis may be attributed to the possible inclusion of uninformative variables in the PCA and OPLS models, masking the information of the few metabolites that reached the significant level after univariate analysis [48].

Overall, the major limitation of the current study is the use of an index-based characterization of NAFLD, which may not predict accurately the presence of NASH [19]. Currently, the ‘gold standard’ for the diagnosis of NASH is liver biopsy [49], still it’s not convenient for prospective studies, and is invasive for the participants, prohibiting evaluations during follow-up. Another potential limitation is the small size of the study population. Despite this, several findings reached statistical significance. Finally, participants were elderly Mediterranean individuals at high cardiovascular risk and this may limit the generalizability of the findings to other age-groups.

In summary, our findings indicate that a rearrangement of lipid biosynthesis and circulation, in the liver and consequently the serum of NAFLD patients, can result in a metabolic profile characteristic, or at least reflecting, NAFLD development. However, despite a potential reduction of hepatic lipotoxicity and improved hepatic function in those participants with NAFLD-reversion, side effects of NAFLD pathophysiology may still appear. Thus, the complex metabolic interactions in NAFLD development [50] need to be further explored.

## Additional files

Additional file 1: Detailed metabolomic analysis procedure ([51, 52]). (DOCX 15 kb)

Additional file 2: Estimation of the index of enzyme activities. (DOCX 11 kb)

Additional file 3: Figure S1. Principal component analysis (PCA) model to discriminate between the three baseline states in groups 1, 2 and 3. Model diagnostics (A = 8, R^2^X = 0.651, Q^2^X = 0.264) (DOCX 43 kb)

Additional file 4: Figure S2. Principal component analysis (PCA) model to discriminate between the baseline and final states in group 1 (participants not meeting the NAFLD criteria at baseline and at the end of follow-up). Model diagnostics (A = 6, R^2^X = 0.621, Q^2^X = 0.160) (DOCX 43 kb)

Additional file 5: Figure S3. Principal component analysis (PCA) to discriminate between the baseline and final states in group 2 (suspected cases of NAFLD). Model diagnostics (A = 6, R^2^X = 0.624, Q^2^X = 0.179) (DOCX 39 kb)

Additional file 6: Figure S4. Principal component analysis (PCA) to discriminate between the baseline and final states in group 3 (suspected NAFLD reversion cases). Model diagnostics (A = 6, R^2^X = 0.628, Q^2^X = 0.235) (DOCX 42 kb)

Additional file 7: Figure S5. Heatmap representing individual metabolomic features obtained from the comparisons between the three baseline states in groups 1, 2 and 3.Heatmapcolor codes for log_2_ (fold-change) and ANOVA and Tukey’s Honestly Significance Difference post hoc test *p*-values are indicated at the bottom of the Figure. Darker green and red colors indicate higher drops or elevations of the metabolite levels in every comparison. Grey lines correspond to significant fold-changes of individual metabolites, darker grey colors have been used to highlight higher significances (*p* < 0.05, *p* < 0.01 or *p* < 0.001). Group 1 Participants did not meet NAFLD criteria at the baseline and during the follow-up. Group 2 Participants did not meet NAFLD criteria at baseline, but met NAFLD criteria during the follow-up. Group 3 Participants met NAFLD criteria at baseline, but not during the follow-up. Abbreviations: AA, amino acids; SFA, saturated fatty acids; PUFA, polyunsaturated fatty acids; MUFA, monounsaturated fatty acids; NAE, N-acyl ethanolamines; FFAox, free fatty acid oxidised; AC, acyl carnitines; PC, phosphatidylcholine; LPC, lysophosphatidylcholine; PE, phatidylethanolamine; LPE, lysophosphatidylethanolamine; PI, phatidylinositols; LPI, lysophosphatidylinositols; Cer, ceramides; SM, sphingomyelin; ChoE, cholesteryl esters; Chol, cholesterol; TAG, triacylglycerols; DAG, diacylglycerols, BA, bile acids; CMH, monohexosylceramides (DOCX 870 kb)

Additional file 8: Figure S6. Heatmap representing individual metabolomic features obtained from all the comparisons made between the final and the baseline states in groups 1, 2 and 3. Heatmapcolor codes for log_2_ (fold-change) and Student’s t-test *p*-values are indicated at the bottom of the Figure. Darker green and red colors indicate higher drops or elevations of the metabolite levels in every comparison. Grey lines correspond to significant fold-changes of individual metabolites, darker grey colors have been used to highlight higher significances (*p* < 0.05, *p* < 0.01 or *p* < 0.001). Group 1 Participants did not meet NAFLD criteria at the baseline and during the follow-up. Group 2 Participants did not meet NAFLD criteria at baseline, but met NAFLD criteria during the follow-up. Group 3 Participants met NAFLD criteria at baseline, but not during the follow-up. Abbreviations: AA, amino acids; SFA, saturated fatty acids; PUFA, polyunsaturated fatty acids; MUFA, monounsaturated fatty acids; NAE, N-acyl ethanolamines; FFAox, free fatty acid oxidised; AC, acyl carnitines; PC, phosphatidylcholine; LPC, lysophosphatidylcholine; PE, phatidylethanolamine; LPE, lysophosphatidylethanolamine; PI, phatidylinositols; LPI, lysophosphatidylinositols; Cer, ceramides; SM, sphingomyelin; ChoE, cholesteryl esters; Chol, cholesterol; TAG, triacylglycerols; DAG, diacylglycerols, BA, bile acids; CMH, monohexosylceramides (DOCX 1038 kb)

Additional file 9: Figure S7. Volcano plot [−log_10_ (*p*-value) vs. log_2_ (fold-change)] for the comparison between the baseline and final states in group 1 (not meeting the NAFLD criteria at baseline and during the follow-up).Abbreviations: AA, amino acids; SFA, saturated fatty acids; PUFA, polyunsaturated fatty acids; MUFA, monounsaturated fatty acids; NAE, N-acyl ethanolamines; FFAox, free fatty acid oxidised; AC, acyl carnitines; PC, phosphatidylcholine; LPC, lysophosphatidylcholine; PE, phatidylethanolamine; LPE, lysophosphatidylethanolamine; PI, phatidylinositols; LPI, lysophosphatidylinositols; Cer, ceramides; SM, sphingomyelin; ChoE, cholesteryl esters; Chol, cholesterol; TAG, triacylglycerols; DAG, diacylglycerols, BA, bile acids; CMH, monohexosylceramides (DOCX 50 kb)

Additional file 10: Figure S8. Volcano plot [−log_10_ (*p*-value) vs. log_2_ (fold-change)] for the comparison between the baseline and final states in group 2 (suspected cases of NAFLD). Abbreviations: AA, amino acids; SFA, saturated fatty acids; PUFA, polyunsaturated fatty acids; MUFA, monounsaturated fatty acids; NAE, N-acyl ethanolamines; FFAox, free fatty acid oxidised; AC, acyl carnitines; PC, phosphatidylcholine; LPC, lysophosphatidylcholine; PE, phatidylethanolamine; LPE, lysophosphatidylethanolamine; PI, phatidylinositols; LPI, lysophosphatidylinositols; Cer, ceramides; SM, sphingomyelin; ChoE, cholesteryl esters; Chol, cholesterol; TAG, triacylglycerols; DAG, diacylglycerols, BA, bile acids; CMH, monohexosylceramides (DOCX 52 kb)

Additional file 11: Figure S9. Volcano plot [−log_10_ (*p*-value) vs. log_2_ (fold-change)] for the comparison between the baseline and final states in group 3 (suspected NAFLD reversion cases). Abbreviations: AA, amino acids; SFA, saturated fatty acids; PUFA, polyunsaturated fatty acids; MUFA, monounsaturated fatty acids; NAE, N-acyl ethanolamines; FFAox, free fatty acid oxidised; AC, acyl carnitines; PC, phosphatidylcholine; LPC, lysophosphatidylcholine; PE, phatidylethanolamine; LPE, lysophosphatidylethanolamine; PI, phatidylinositols; LPI, lysophosphatidylinositols; Cer, ceramides; SM, sphingomyelin; ChoE, cholesteryl esters; Chol, cholesterol; TAG, triacylglycerols; DAG, diacylglycerols, BA, bile acids; CMH, monohexosylceramides (DOCX 50 kb)

## Abbreviations

BCAAs: branched chainamino acids
Cer: ceramide
ChoE: cholesterylesters
Chol: cholesterol
D5D: delta-5-desaturase
D6D: delta-6-desaturase
Elovl6: elongase 6
FAs: fatty acids
HSI: hepatic steatosis index
LC-MS/MS: liquid chromatography tandem massspectrometry
LPC: lysophosphatidylcholine
LPE: lysophosphatidylethanolamine
MUFAs: monounsaturated fatty acids
NAFLD: Non-alcoholic fatty liver disease
NASH: non-alcoholic steatohepatitis
NEFAs: non esterified fatty acids
OPLS: orthogonal partial least-squares to latent structures
PC: phosphatidylcholine
PCA: principal component analysis
PE: phosphatidylethanolamine
PUFAs: polyunsaturated fatty acids
SCD1: stearoyl-CoA desaturase 1
SM: sphingomyelin

## References

[1] Vernon G, Baranova A, Younossi ZM (2011). Systematic review: the epidemiology and natural history of non-alcoholic fatty liver disease and non-alcoholic steatohepatitis in adults. Aliment Pharmacol Ther.

[2] Bellentani S, Scaglioni F, Marino M, Bedogni G (2010). Epidemiology of non-alcoholic fatty liver disease. Dig Dis.

[3] Chalasani N, Younossi Z, Lavine JE, Diehl AM, Brunt EM, Cusi K (2012). The diagnosis and management of non-alcoholic fatty liver disease: practice guideline by the American Association for the Study of Liver Diseases, American College of Gastroenterology, and the American Gastroenterological Association. Hepatology.

[4] Rafiq N, Bai C, Fang Y, Srishord M, McCullough A, Gramlich T (2009). Long-term follow-up of patients with nonalcoholic fatty liver. Clin Gastroenterol Hepatol.

[5] Dowman JK, Tomlinson JW, Newsome PN (2010). Pathogenesis of non-alcoholic fatty liver disease. QJM.

[6] Youssef W, McCullough AJ (2002). Diabetes mellitus, obesity, and hepatic steatosis. Semin Gastrointest Dis.

[7] Vacca M, Allison M, Griffin JL, Vidal-Puig A (2015). Fatty acid and glucose sensors in hepatic lipid metabolism: implications in NAFLD. Semin Liver Dis.

[8] Puri P, Baillie RA, Wiest MM, Mirshahi F, Choudhury J, Cheung O (2007). A lipidomic analysis of nonalcoholic fatty liver disease. Hepatology.

[9] Anjani K, Lhomme M, Sokolovska N, Poitou C, Aron-Wisnewsky J, Bouillot JL (2015). Circulating phospholipid profiling identifies portal contribution to NASH signature in obesity. J Hepatol.

[10] Puri P, Wiest MM, Cheung O, Mirshahi F, Sargeant C, Min HK (2009). The plasma lipidomic signature of nonalcoholic steatohepatitis. Hepatology.

[11] Park H, Hasegawa G, Shima T, Fukui M, Nakamura N, Yamaguchi K (2010). The fatty acid composition of plasma cholesteryl esters and estimated desaturase activities in patients with nonalcoholic fatty liver disease and the effect of long-term ezetimibe therapy on these levels. Clin Chim Acta.

[12] Walle P, Takkunen M, Männistö V, Vaittinen M, Lankinen M, Kärjä V (2016). Fatty acid metabolism is altered in non-alcoholic steatohepatitis independent of obesity. Metabolism.

[13] Kalhan SC, Guo L, Edmison J, Dasarathy S, McCullough AJ, Hanson RW (2011). Plasma Metabolomic profile in non-alcoholic fatty liver disease. Metabolism.

[14] Lake AD, Novak P, Shipkova P, Aranibar N, Robertson DG, Reily MD (2015). Branched chain amino acid metabolism profiles in progressive human nonalcoholic fatty liver disease. Amino Acids.

[15] Gibney MJ, Walsh M, Brennan L, Roche HM, German B, van Ommen B (2005). Metabolomics in human nutrition: opportunities and challenges. Am J Clin Nutr.

[16] Hollywood K, Brison DR, Goodacre R (2006). Metabolomics: current technologies and future trends. Proteomics.

[17] Estruch R, Ros E, Salas-Salvadó J, Covas MI, Corella D, Arós F (2013). Primary prevention of cardiovascular disease with a Mediterranean diet. N Engl J Med.

[18] Martínez-González MÁ, Corella D, Salas-Salvadó J, Ros E, Covas MI, Fiol M (2012). Cohort profile: design and methods of the PREDIMED study. Int J Epidemiol.

[19] Lee JH, Kim D, Kim HJ, Lee CH, Yang JI, Kim W (2010). Hepatic steatosis index: a simple screening tool reflecting nonalcoholic fatty liver disease. Dig Liver Dis.

[20] Baker M (2011). Metabolomics: from small molecules to big ideas. Nat Methods.

[21] Duportet X, Aggio RS. Carneiro S, Villas-Boas SG. The biological interpretation of metabolomics data can be misled by the extraction method used. Metabolomics Metabolomics 2012;8:410–421.

[22] Barr J, Vázquez-Chantada M, Alonso C, Pérez-Cormenzana M, Mayo R, Galán A (2010). Liquid chromatography-mass spectrometry-based parallel metabolic profiling of human and mouse model serum reveals putative biomarkers associated with the progression of nonalcoholic fatty liver disease. J Proteome Res.

[23] Barr J, Caballería J, Martínez-Arranz I, Domínguez-Díez A, Alonso C, Muntané J (2012). Obesity-dependent metabolic signatures associated with nonalcoholic fatty liver disease progression. J Proteome Res.

[24] van der Kloet FM, Bobeldijk I, Verheij ER, Jellema RH (2009). Analytical error reduction using single point calibration for accurate and precise metabolomic phenotyping. J Proteome Res.

[25] Peter A, Cegan A, Wagner S, Elcnerova M, Königsrainer A, Königsrainer I (2011). Relationships between hepatic stearoyl-CoA desaturase-1 activity and mRNA expression with liver fat content in humans. AJP Endocrinol Metab.

[26] Jolliffe IT. Principal component analysis. Springer. 2nd ed. New York: 2002.

[27] Wiklund S, Johansson E, Sjöström L, Mellerowicz EJ, Edlund U, Shockcor JP (2007). Visualization of GC/TOF-MS-based metabolomics data for identification of biochemically interesting compounds using OPLS class models. Anal Chem.

[28] Bylesjö M, Rantalainen M, Cloarec O, Nicholson JK, Holmes E, Trygg J (2006). OPLS discriminant analysis: combining the strengths of PLS-DA and SIMCA classification. J Chemom.

[29] Almatov KT, Abdullaev GR (2016). Alterations of phospholipids synthesis in mitochondrial membrane of the liver cells in the dynamics of chronic emotional painful stress development. Am J Biochem.

[30] Jenkins RW, Canals D, Hannun YA (2009). Roles and regulation of secretory and lysosomal acid sphingomyelinase. Cell Signal.

[31] Kakazu E, Ueno Y, Kondo Y, Inoue J, Ninomiya M, Kimura O (2011). Plasma L-cystine/L-glutamate imbalance increases tumor necrosis factor-alpha from CD14+ circulating monocytes in patients with advanced cirrhosis. PLoS One.

[32] Chen Y, Varghese Z, Ruan XZ (2014). The molecular pathogenic role of inflammatory stress in dysregulation of lipid homeostasis and hepatic steatosis. Genes Dis.

[33] Garcia-Ruiz C, Mato JM, Vance D, Kaplowitz N, Fernández-Checa JC (2015). Acid sphingomyelinase-ceramide system in steatohepatitis: a novel target regulating multiple pathways. J Hepatol.

[34] Han MS, Park SY, Shinzawa K, Kim S, Chung KW, Lee JH (2008). Lysophosphatidylcholine as a death effector in the lipoapoptosis of hepatocytes. J Lipid Res.

[35] Kakisaka K, Cazanave SC, Fingas CD, Guicciardi ME, Bronk SF, Werneburg NW (2012). Mechanisms of lysophosphatidylcholine-induced hepatocyte lipoapoptosis. Am J Physiol Gastrointest Liver Physiol.

[36] Barceló F, Perona JS, Prades J, Funari SS, Gomez-Gracia E, Conde M (2009). Mediterranean-style diet effect on the structural properties of the erythrocyte cell membrane of hypertensive patients: the prevencion con dieta mediterranea study. Hypertension.

[37] Li Z, Agellon LB, Allen TM, Umeda M, Jewell L, Mason A (2006). The ratio of phosphatidylcholine to phosphatidylethanolamine influences membrane integrity and steatohepatitis. Cell Metab.

[38] Landschulz KT, Jump DB, MacDougald OA, Lane MD (1994). Transcriptional control of the stearoyl-CoA desaturase-1 gene by polyunsaturated fatty acids. Biochem Biophys Res Commun.

[39] Ntambi JM, Miyazaki M (2004). Regulation of stearoyl-CoA desaturases and role in metabolism. Prog Lipid Res.

[40] Chong MF, Hodson L, Bickerton AS, Roberts R, Neville M, Karpe F, et al. Parallel activation of de novo lipogenesis and stearoyl-CoA desaturase activity after 3d of high-carbohydrate feeding. Am J Clin Nutr. 2008;87:817–23. 10.1093/ajcn/87.4.817 18400702

[41] Sampath H, Miyazaki M, Dobrzyn A, Ntambi JM (2007). Stearoyl-CoA desaturase-1 mediates the pro-lipogenic effects of dietary saturated fat. J Biol Chem.

[42] Emken EA (1994). Metabolism of dietary stearic acids in human acid relative to other fatty acids in human subjects. Am J Clin Nutr.

[43] Matsuzaka T, Atsumi A, Matsumori R, Nie T, Shinozaki H, Suzuki-Kemuriyama N (2012). Elovl6 promotes nonalcoholic steatohepatitis. Hepatology.

[44] Sanders FW, Griffin JL (2016). De novo lipogenesis in the liver in health and disease: more than just a shunting yard for glucose. Biol Rev.

[45] Spadaro L, Magliocco O, Spampinato D, Piro S, Oliveri C, Alagona C (2008). Effects of n-3 polyunsaturated fatty acids in subjects with nonalcoholic fatty liver disease. Dig Liver Dis.

[46] Lee RG, Kelley KL, Sawyer JK, Farese RV, Parks JS, Rudel LL (2004). Plasma cholesteryl esters provided by lecithin: cholesterol acyltransferase and acyl-coenzyme a:cholesterol acyltransferase 2 have opposite atherosclerotic potential. Circ Res.

[47] Araya J, Rodrigo R, Videla LA, Thielemann L, Orellana M, Pettinelli P (2004). Increase in long-chain polyunsaturated fatty acid n - 6/n - 3 ratio in relation to hepatic steatosis in patients with non-alcoholic fatty liver disease. Clin Sci (Lond).

[48] Saccenti E, Hoefsloot HJ, Smilde A, Westerhuis J, Hendriks M (2014). Reflections on univariate and multivariate analysis of metabolomics data. Metabolomics.

[49] Byrne CD, Olufadi R, Bruce KD, Cagampang FR, Ahmed MH (2009). Metabolic disturbances in non-alcoholic fatty liver disease. Clin Sci (Lond).

[50] Chiappini F, Coilly A, Kadar H, Gual P, Tran A, Desterke C (2017). Metabolism dysregulation induces a specific lipid signature of nonalcoholic steatohepatitis in patients. Sci Rep.

[51] Grumbach ES, Wheat TE. Mazzeo JR. A novel method for the analysis of amino acids. www.waters.com

[52] Martínez-Arranz I, Mayo R, Pérez-Cormenzana M, Mincholé I, Salazar L, Alonso C (2015). Enhancing metabolomics research through data mining. J Proteome.

