# Supplementary material for: Serum metabolites in non-alcoholic fatty-liver disease development or reversion; a targeted metabolomic approach within the PREDIMED trial
Source: Nutr Metab (Lond). 2017 Sep 2;14:58. doi: 10.1186/s12986-017-0213-3 (PMC5581927; doi:10.1186/s12986-017-0213-3)
Supplement: Supplementary file 1 — Detailed metabolomic analysis procedure ([51, 52]). (DOCX 15 kb) [file 12986_2017_213_MOESM1_ESM.docx]

**Additional file 1**

*Fatty acyls, bile acids, steroids and lysoglycerophospholipids profiling*. Proteins were precipitated from the defrosted serum samples by adding 4 volumes of methanol in 1.5 mL tubes, at room temperature. To facilitate quantization and comparison, the methanol used for extraction was spiked with metabolites not detected in unspiked human-serum extracts. After brief vortex-mixing, samples were incubated overnight, at -20 °C. Supernatants were collected after centrifugation at 18,000 x g for 15 min, dried and reconstituted in methanol for analysis.

*Glycerolipids, cholesteryl esters, sphingolipids and glycerolphospholipids profiling.* Serum extracts were mixed with sodium chloride (50 mM) and chloroform/methanol (2:1) in 1.5 mL tubes at room temperature. The extraction solvent was spiked with metabolites not detected in unspiked human-serum extracts. After brief vortex-mixing, samples were incubated for 1 hr, at -20 °C. After centrifugation at 16,000 x g for 15 min, the organic phase was collected and the solvent removed. The dried extracts were reconstituted in acetronitrile/isopropanol (1:1), centrifuged (18,000 x g for 5 min), and analyzed.

*Amino acids profiling.* Ten μl aliquots of the extracts prepared for platform (1) were transferred to microtubes and derivatized for amino acid analysis [24].

**Pre-processing of metabolomics data**

Data pre-processing generated a list of chromatographic peak areas for the metabolites detected. An approximated linear detection range was defined for each identified metabolite assuming similar detector response levels for all metabolites belonging to a given chemical class, represented by a single standard compound. Data points lying outside their corresponding linear detection range were replaced with missing values, and metabolites for which more than 30% of data points were found outside their corresponding linear detection range, were not analyzed.

Data normalization was performed following the procedure described by Martínez-Arranz et al [25]. Once normalized, the dimensionality of the complex data set was reduced to enable easy visualization of any metabolic clustering of the different groups of samples.
