# Supplementary material for: Serum metabolites in non-alcoholic fatty-liver disease development or reversion; a targeted metabolomic approach within the PREDIMED trial
Source: Nutr Metab (Lond). 2017 Sep 2;14:58. doi: 10.1186/s12986-017-0213-3 (PMC5581927; doi:10.1186/s12986-017-0213-3)
Supplement: Supplementary file 2 — Estimation of the index of enzyme activities. (DOCX 11 kb) [file 12986_2017_213_MOESM2_ESM.docx]

**Additional file 2**

The index of stearoyl-CoA desaturase 1 (SCD1) activity as the ratio of 16:1n-7 to 16:0 (palmitoleic/palmitic), and 18:1n-9 to 18:0 (oleic/stearic); The index of elongase-6 activity as the ratio of 18:0 to 16:0 (stearic/palmitic); The index of elongase-5 activity as 18:1n-7/16:1n-7 (vaccenic/palmitoleic); The index of delta-5-desaturase (D5D) activity as the ratio of 20:4n-6 to 20:3n-6 (arachidonic/dihomo gamma linolenic acid); The index of delta-6-desaturase (D6D) activity as the ratio of 18:3 to 18:2, 18:3n-6 to 18:2n-6 (gamma-linolenic acid/linoleic acid) and 18:4n-3 to 18:3n-3 (stearidonic acid/α-linolenic acid). The index of de novo lipogenesis was assessed based on the 16:0 to 18:2n-6 (palmitic/linoleic acid) ratio. The index of phosphatidylethanolamine methyltransferase activity was also evaluated by calculating ratio of phosphatidylcholine to phosphatidylethanolamine (PC/PE). The activity index of diacylglycerol acyltransferase was assessed by the ratio TAG/DAG. In addition, the MUFA+PUFA to saturated FA and PUFA to MUFA ratios were used to estimate overall index of desaturase activity. The index of phospholipase-A2 activity [LPC/PC (lysophosphatidylcholine/phosphatidylcholine) and LPE/PE (lysophosphatidylethanolamine/phosphatidylethanolamine)], acid sphingomyelinase activity [Cer/SM (ceramide/sphingomyelin)] and lecithin:cholesterol acyltransferase [ChoE/Chol (cholesteryl esters/cholesterol)] were also assessed.
