# Supplementary figures and images for: Serum metabolites in non-alcoholic fatty-liver disease development or reversion; a targeted metabolomic approach within the PREDIMED trial
Source: Nutr Metab (Lond). 2017 Sep 2;14:58. doi: 10.1186/s12986-017-0213-3 (PMC5581927; doi:10.1186/s12986-017-0213-3)

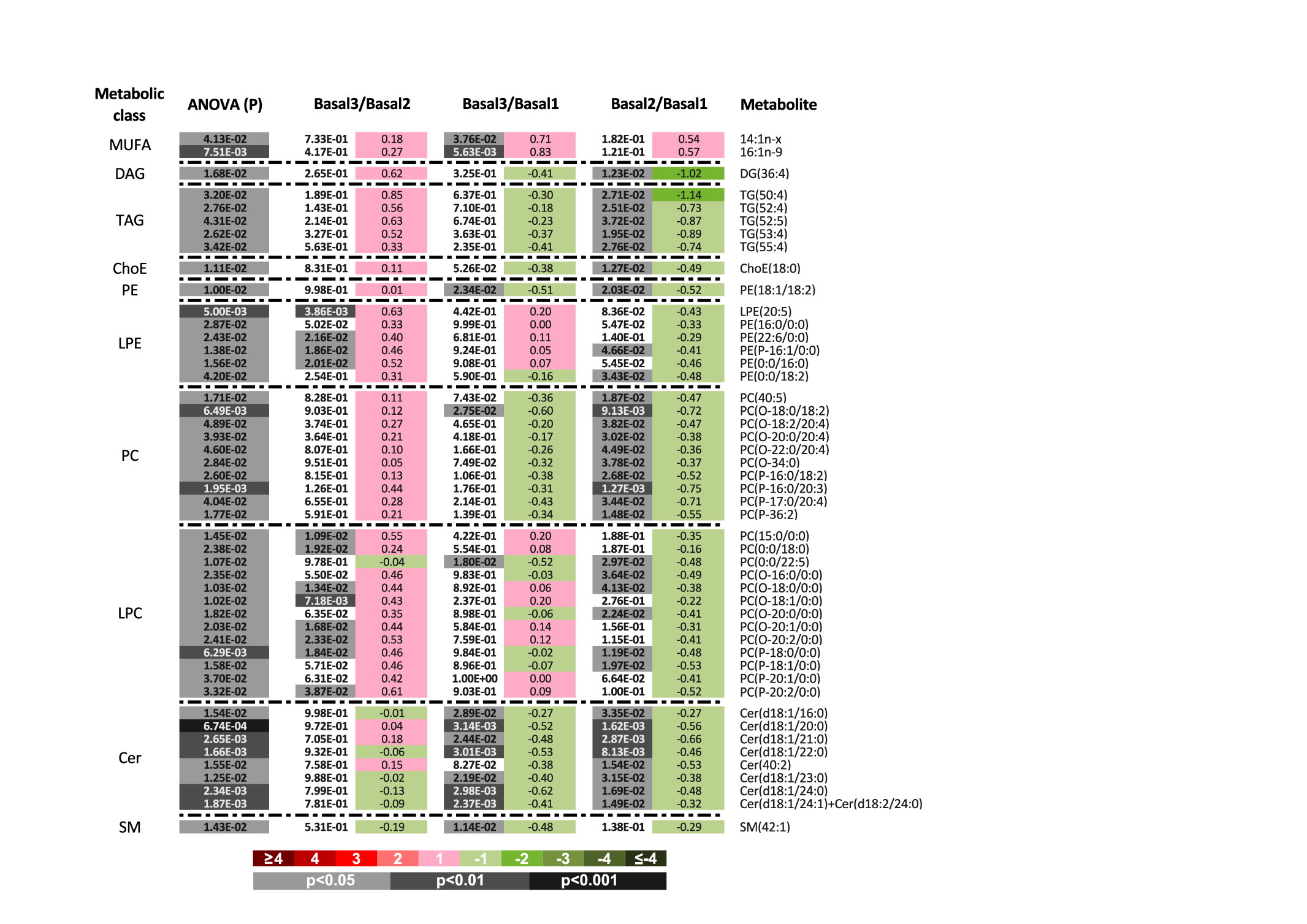

Supplement: Supplementary file 7 — Heatmap representing individual metabolomic features obtained from the comparisons between the three baseline states in groups 1, 2 and 3.Heatmapcolor codes for log2 (fold-change) and ANOVA and Tukey’s Honestly Significance Difference post hoc test p-values are indicated at the bottom of the Figure. Darker green and red colors indicate higher drops or elevations of the metabolite levels in every comparison. Grey lines correspond to significant fold-changes of individual metabolites, darker grey colors have been used to highlight higher significances (p < 0.05, p < 0.01 or p < 0.001). Group 1 Participants did not meet NAFLD criteria at the baseline and during the follow-up. Group 2 Participants did not meet NAFLD criteria at baseline, but met NAFLD criteria during the follow-up. Group 3 Participants met NAFLD criteria at baseline, but not during the follow-up. Abbreviations: AA, amino acids; SFA, saturated fatty acids; PUFA, polyunsaturated fatty acids; MUFA, monounsaturated fatty acids; NAE, N-acyl ethanolamines; FFAox, free fatty acid oxidised; AC, acyl carnitines; PC, phosphatidylcholine; LPC, lysophosphatidylcholine; PE, phatidylethanolamine; LPE, lysophosphatidylethanolamine; PI, phatidylinositols; LPI, lysophosphatidylinositols; Cer, ceramides; SM, sphingomyelin; ChoE, cholesteryl esters; Chol, cholesterol; TAG, triacylglycerols; DAG, diacylglycerols, BA, bile acids; CMH, monohexosylceramides (DOCX 870 kb) [file 12986_2017_213_MOESM7_ESM.docx]

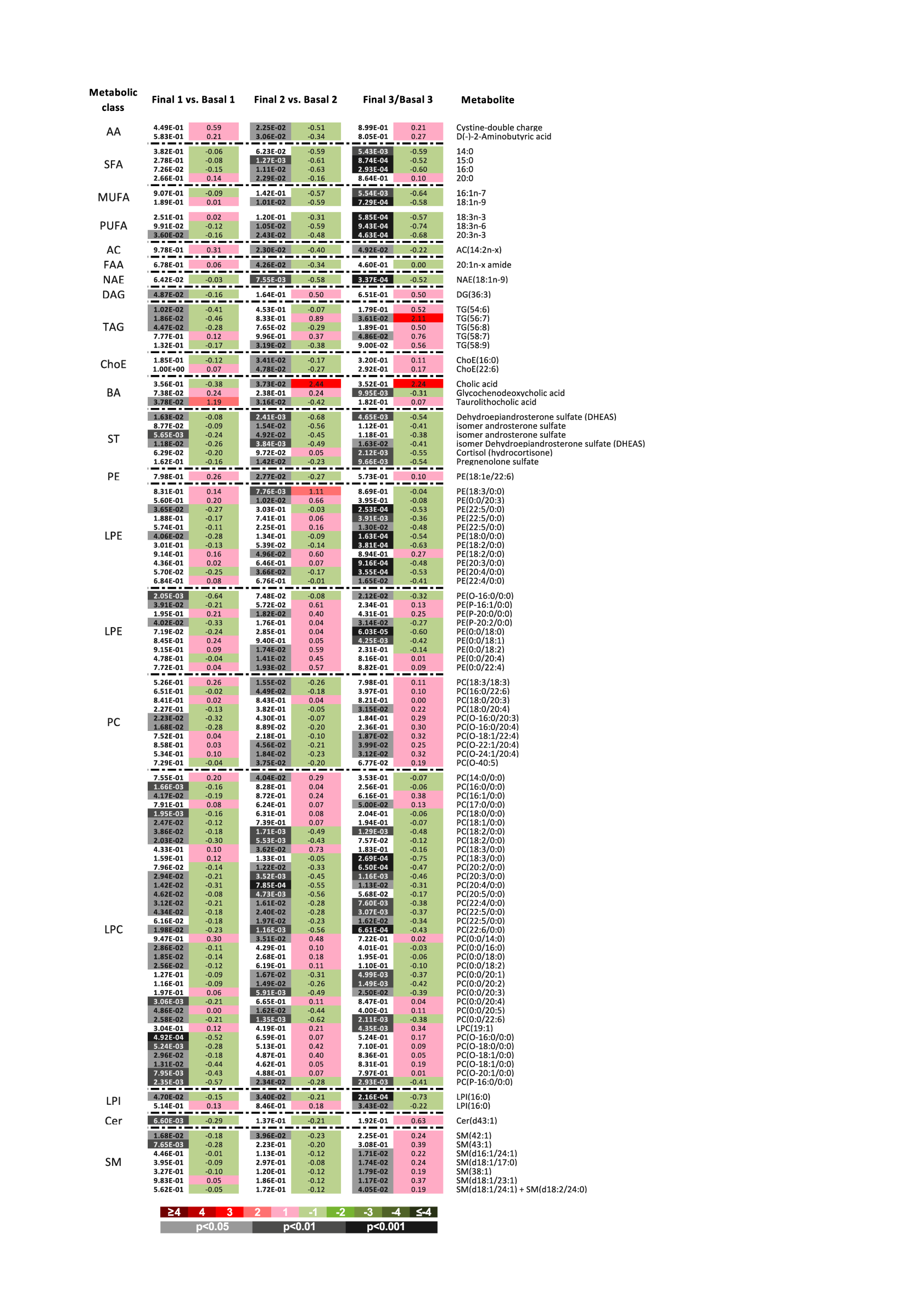

Supplement: Supplementary file 8 — Heatmap representing individual metabolomic features obtained from all the comparisons made between the final and the baseline states in groups 1, 2 and 3. Heatmapcolor codes for log2 (fold-change) and Student’s t-test p-values are indicated at the bottom of the Figure. Darker green and red colors indicate higher drops or elevations of the metabolite levels in every comparison. Grey lines correspond to significant fold-changes of individual metabolites, darker grey colors have been used to highlight higher significances (p < 0.05, p < 0.01 or p < 0.001). Group 1 Participants did not meet NAFLD criteria at the baseline and during the follow-up. Group 2 Participants did not meet NAFLD criteria at baseline, but met NAFLD criteria during the follow-up. Group 3 Participants met NAFLD criteria at baseline, but not during the follow-up. Abbreviations: AA, amino acids; SFA, saturated fatty acids; PUFA, polyunsaturated fatty acids; MUFA, monounsaturated fatty acids; NAE, N-acyl ethanolamines; FFAox, free fatty acid oxidised; AC, acyl carnitines; PC, phosphatidylcholine; LPC, lysophosphatidylcholine; PE, phatidylethanolamine; LPE, lysophosphatidylethanolamine; PI, phatidylinositols; LPI, lysophosphatidylinositols; Cer, ceramides; SM, sphingomyelin; ChoE, cholesteryl esters; Chol, cholesterol; TAG, triacylglycerols; DAG, diacylglycerols, BA, bile acids; CMH, monohexosylceramides (DOCX 1038 kb) [file 12986_2017_213_MOESM8_ESM.docx]
