# Supplementary material for: Serum metabolites in non-alcoholic fatty-liver disease development or reversion; a targeted metabolomic approach within the PREDIMED trial
Source: Nutr Metab (Lond). 2017 Sep 2;14:58. doi: 10.1186/s12986-017-0213-3 (PMC5581927; doi:10.1186/s12986-017-0213-3)
Supplement: Supplementary file 9 — Volcano plot [−log10 (p-value) vs. log2 (fold-change)] for the comparison between the baseline and final states in group 1 (not meeting the NAFLD criteria at baseline and during the follow-up).Abbreviations: AA, amino acids; SFA, saturated fatty acids; PUFA, polyunsaturated fatty acids; MUFA, monounsaturated fatty acids; NAE, N-acyl ethanolamines; FFAox, free fatty acid oxidised; AC, acyl carnitines; PC, phosphatidylcholine; LPC, lysophosphatidylcholine; PE, phatidylethanolamine; LPE, lysophosphatidylethanolamine; PI, phatidylinositols; LPI, lysophosphatidylinositols; Cer, ceramides; SM, sphingomyelin; ChoE, cholesteryl esters; Chol, cholesterol; TAG, triacylglycerols; DAG, diacylglycerols, BA, bile acids; CMH, monohexosylceramides (DOCX 50 kb) [file 12986_2017_213_MOESM9_ESM.docx]

Figure S7
